# Supplementary material for: Novel Hydrogels Based on the Nano-Emulsion Formulation Process: Development, Rheological Characterization, and Study as a Drug Delivery System
Source: Pharmaceutics. 2024 Jun 14;16(6):812. doi: 10.3390/pharmaceutics16060812 (PMC11207514; doi:10.3390/pharmaceutics16060812)

Novel hydrogels based on nano-emulsions formulation process: development, rheological characterization, and study as drug delivery system

Usama Jamshaid,[a,b] Nicolas Anton,[a,*] Mohamed Elhassan,[a,c] Guillaume Conzatti,[a] Thierry Vandamme [a,*]

[a] *INSERM (French National Institute of Health and Medical Research), UMR 1260, Regenerative Nanomedicine (RNM), FMTS, Université de Strasbourg, F-67000 Strasbourg, France; usamapharma-cy2012@gmail.com (U.J.); mohamed-ahmed.mohamed-elhassan-ahmed@etu.unistra.fr (M.E.); conzatti@unistra.fr (G.C.)*

[b] *Faculty of Pharmacy, The University of Lahore, Lahore 54590, Pakistan*

[c] *Department of Pharmaceutics, Faculty of Pharmacy, University of Gezira, Wad Medani 21111, Sudan*

To whom correspondence should be addressed: Nicolas Anton (nanton@unistra.fr) and Thierry Vandamme (vandamme@unistra.fr)

Table S1: Composition of the representative formulations described in Fig. 1.

	SOR (%)	SOWR (%)	Water (g)	Surfactant (g)	Oil (g)
Liquid state (Fig. 1 (a))	60	44	56	26,4	17,6
Transition state (Fig. 1 (b))	60	52	48	31,2	20,8
Gel state (Fig. 1 (c))	60	60	40	36	24

Statistical analysis for the determination of mean value and standard deviation of the release exponent α within the fitting power law used in Fig. 5 (a), (b), (c), and reported in Fig. 5 (d)

SOR = 40% / SOWR = 40%

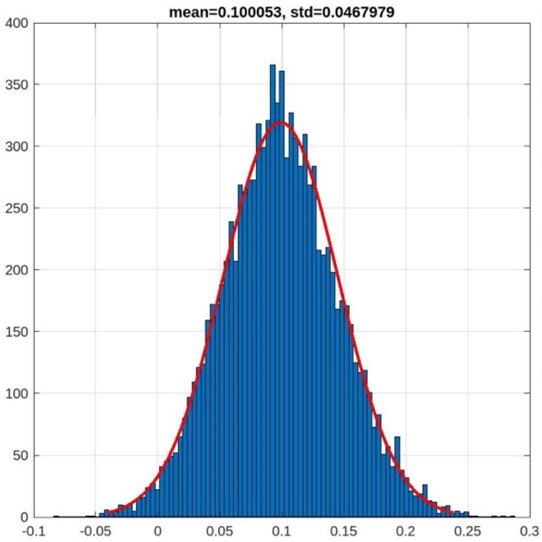

SOR = 40% / SOWR = 50%

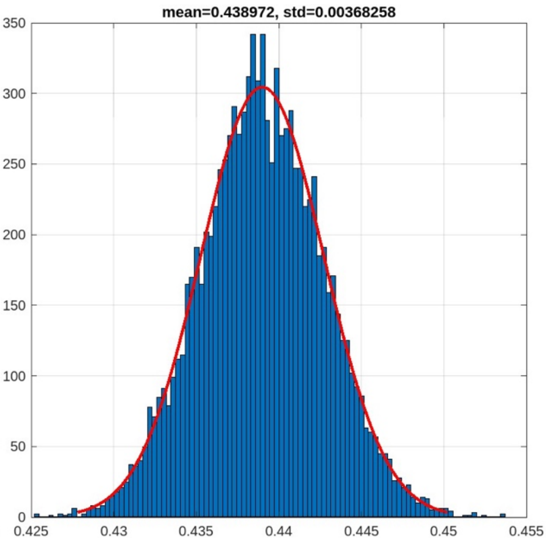

SOR = 40% / SOWR = 40%

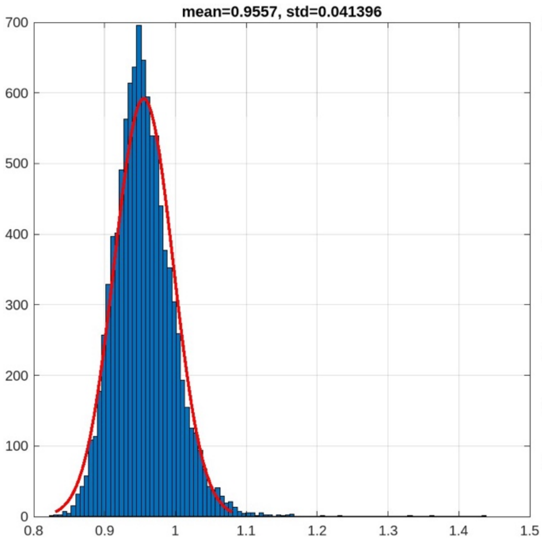

SOR = 40% / SOWR = 50%

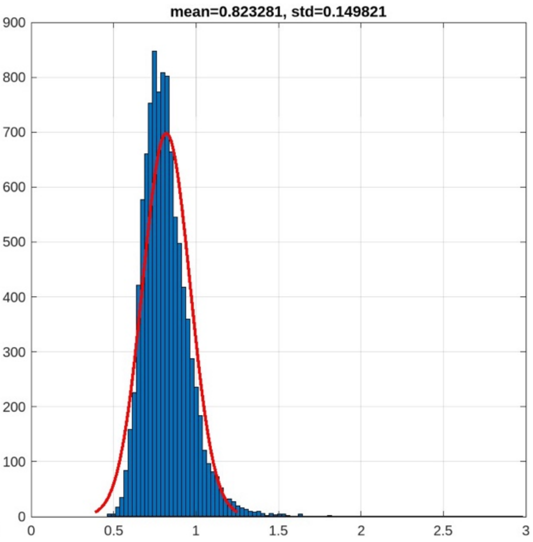

SOR = 60% / SOWR = 40%

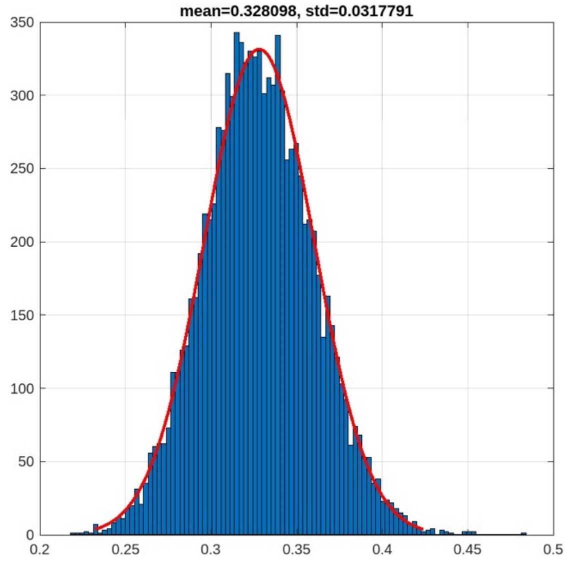

SOR = 60% / SOWR = 50%

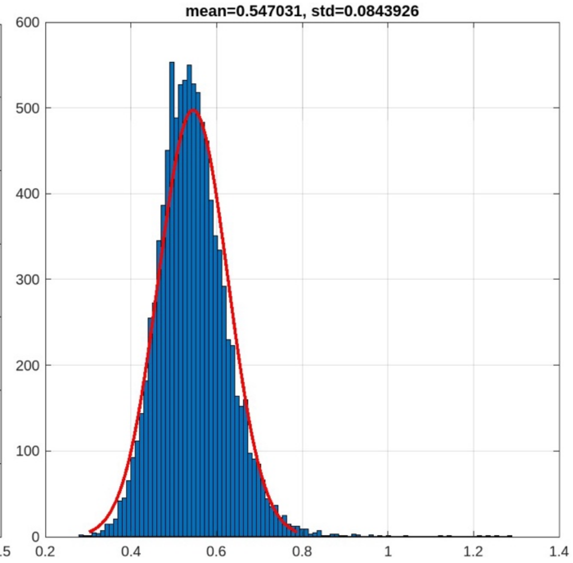

SOR = 60% / SOWR = 60%

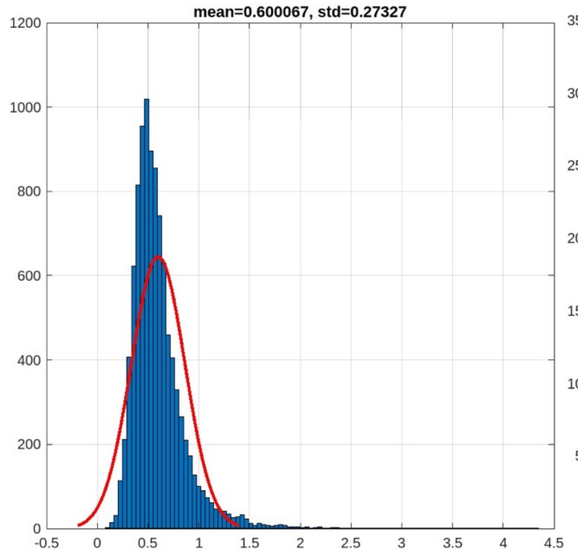

SOR = 60% / SOWR = 70%

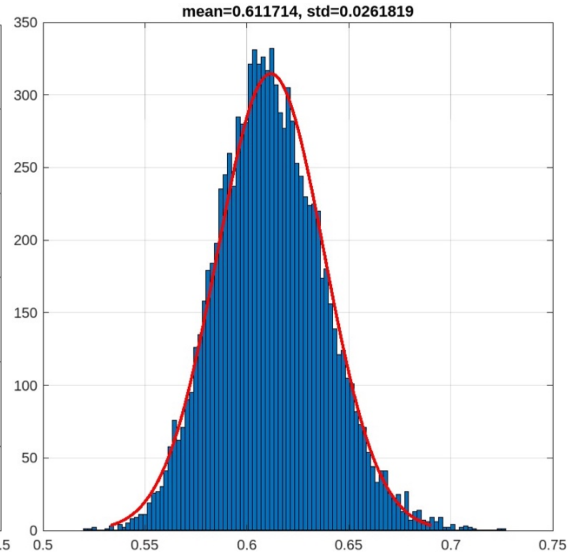

SOR = 70% / SOWR = 40%

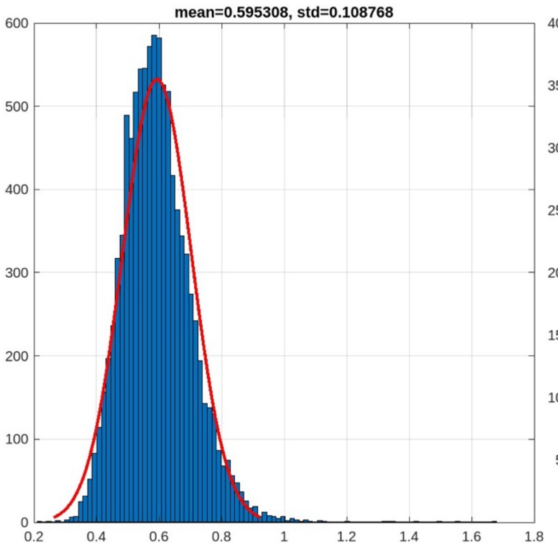

SOR = 70% / SOWR = 50%

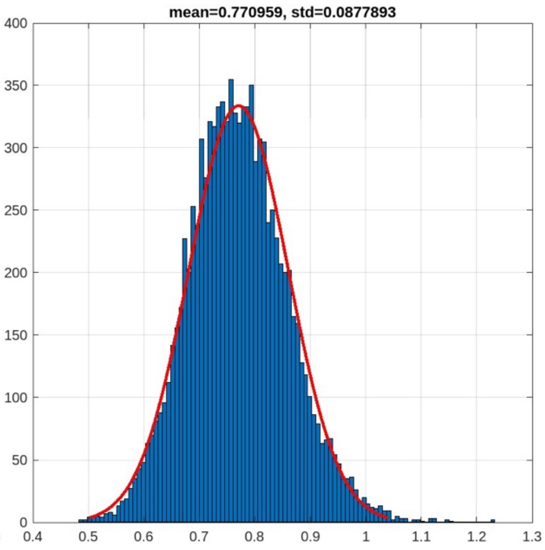

SOR = 70% / SOWR = 60%

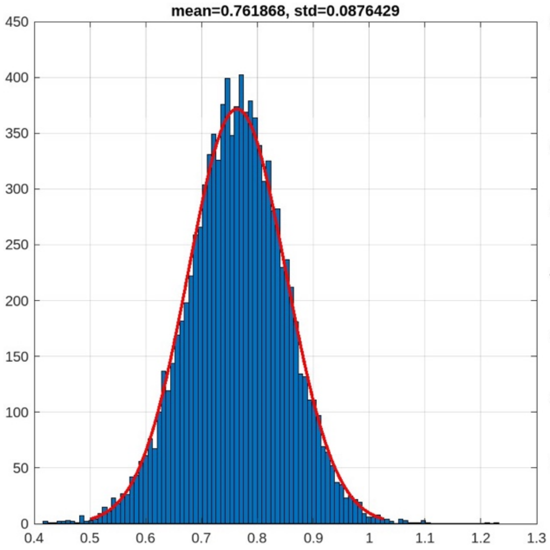

SOR = 70% / SOWR = 70%

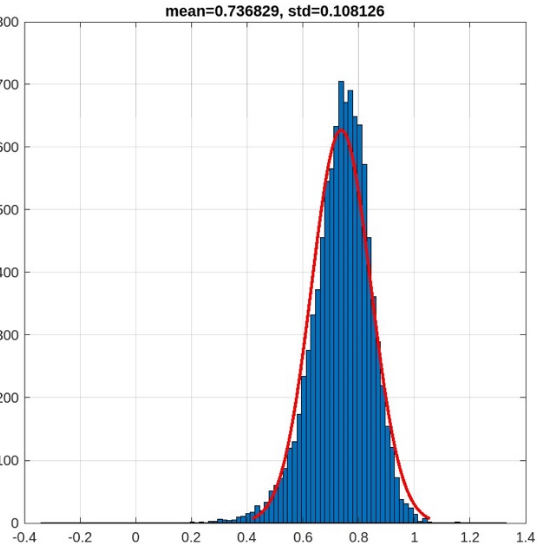

Supplement: Supplementary file 1 [file pharmaceutics-16-00812-s001.zip › pharmaceutics-3039485-supplementary.pdf]
